# Longitudinal three-dimensional photoacoustic imaging reveals hyperoxic perilesional microvasculature associated with keloid recurrence^☆^

**DOI:** 10.1016/j.pacs.2026.100831

**Published:** 2026-04-23

**Authors:** Aiko Makino, Susumu Saito, Ayako Takaya, Tomoko Kosaka, Eiichi Sawaragi, Maria Chiara Munisso, Aya Yoshikawa, Yasufumi Asao, Hiroyuki Sekiguchi, Takayuki Yagi, Naoki Morimoto

**Affiliations:** a Department of Plastic and Reconstructive Surgery, Graduate School of Medicine, Kyoto University, 54 Shogoin-Kawaharacho Sakyo-ku, Kyoto 6068507, Japan; b Department of Technology Development, Luxonus Inc., Kawasaki, Kanagawa, Japan

**Keywords:** Keloid, Photoacoustic Imaging, Three-Dimensional Imaging, Microcirculation, Oxygenation, Longitudinal Evaluation

## Abstract

Keloids undergo repeated cycles of regression and recurrence; however, vascular dynamics associated with recurrence remain poorly understood. In this prospective time-series study, we investigated microvascular changes in keloids using longitudinal three-dimensional photoacoustic imaging combined with ultrasonography. Adult patients with trunk or extremity keloids receiving intralesional triamcinolone treatment underwent imaging at four time points over a 6-month period. Vascular morphology and oxygenation were evaluated in superficial and subdermal layers using dual-wavelength near-infrared illumination and relative S-factor values referenced to peri-keloid regions. Six keloids in five patients were analyzed. Regressive lesions demonstrated hypoxic superficial microvessels disconnected from hyperoxic subdermal vessels, whereas recurrent lesions showed emergence of hyperoxic superficial microvessels continuous with hyperoxic subdermal venous networks. Persistent subdermal venous hyperoxia was observed irrespective of clinical status. Combined three-dimensional and longitudinal photoacoustic imaging enabled spatiotemporal identification of activity-dependent microvascular alterations associated with keloid recurrence, supporting a potential arteriovenous shunting mechanism.

## Graphical Abstract

**Figure:**
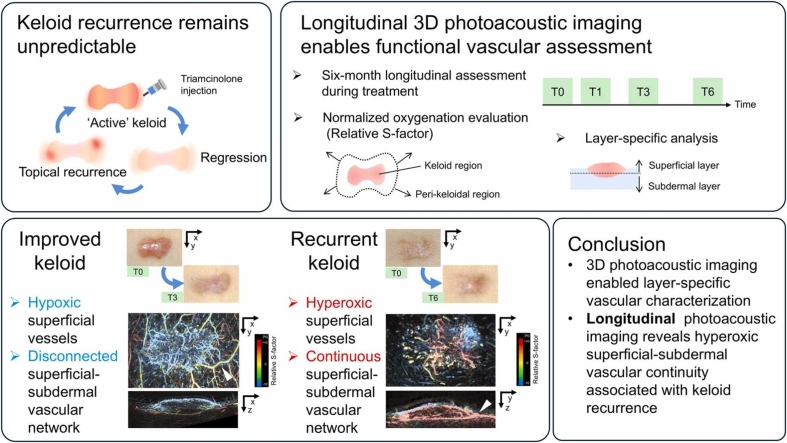

## 1 Introduction

Keloids are hyperplastic skin lesions characterized by excessive collagen deposition and persistent fibroproliferative activity. Clinically, they present as raised, erythematous lesions that extend beyond the boundaries of the original wound [1]. Common symptoms, including pruritus, tightness, and pain, cause substantial physical discomfort and negatively affect patients’ quality of life. Treatment options range from conservative to surgical approaches; however, recurrence rates exceed 50% following surgical excision alone [2] and remain high, ranging from 6% to 32.7% even when combined with adjuvant radiation therapy [3], [4]. Conservative treatments include pressure therapy, silicone sheets, intralesional corticosteroid injections or films, and oral antiallergic drugs [5]. Among these modalities, intralesional corticosteroid injections are the most widely used; nevertheless, recurrence rates of approximately 33% within 1 year and up to 50% within 5 years have been reported [6]. In many patients, keloids undergo repeated cycles of temporary regression, local recurrence, progressive lesion enlargement, symptom exacerbation, and repeated corticosteroid injections. As keloids enlarge, a greater surface area requires treatment, resulting in multiple injections and substantial procedural pain for patients. Despite the clinical importance of recurrence, the spatiotemporal vascular dynamics associated with keloid relapse remain poorly characterized, and no imaging-based biomarkers currently exist to predict the timing or location of recurrence during longitudinal follow-up.

Abnormal vascular patterns have been increasingly recognized in keloids. Clinically, an association between keloid severity and hypertension has been reported [7]. Histopathological studies have demonstrated a reduced vascular density within the keloid core together with superficial perikeloidal microvessel proliferation, luminal narrowing, and pronounced inflammatory changes, suggesting that impaired microvascular circulation contributes to disease progression [8], [9], [10]. However, conventional histological and imaging approaches provide only static observations, and the hemodynamic alterations that precede or accompany local keloid recurrence in vivo remain largely unexplored.

Recently, photoacoustic imaging (PAI) has emerged as a promising hybrid optical–ultrasound modality for functional vascular imaging [11], [12], [13]. This technique is based on the photoacoustic effect, in which absorbed optical energy generates ultrasonic waves, enabling visualization of blood vessels with optical contrast at ultrasonic resolution [14], [15]. Importantly, PAI allows simultaneous assessment of vascular morphology and hemoglobin oxygenation, enabling functional evaluation of tissue hemodynamics. In particular, in studies of cutaneous malignancies and chronic inflammatory skin diseases, PAI, along with higher-resolution photoacoustic microscopy (PAM), has increasingly been used to detect vascular abnormalities [15], [16], [17], [18], [19], [20], [21]. However, most previous studies have relied on two-dimensional imaging and have not adequately characterized dynamic changes in vascular architecture associated with disease activity. Our research group has developed an advanced PAI system incorporating a hemispherical ultrasound detector array, enabling in vivo acquisition of high-resolution three-dimensional (3D) vascular images with a spatial resolution of approximately 0.2 mm [22], [23], [24], [25], [26]. Using this system, we previously conducted clinical observational studies in healthy human trunks and limbs, as well as clinical investigations involving patients with cancer and reconstructive surgical procedures [27], [28]. These studies demonstrated that 3D visualization of vascular architecture and oxygenation enables delineation of subcutaneous perforating arteries and veins and their spatial relationships, information critical for surgical planning. Because PAI is noninvasive and repeatable, it is particularly suitable for longitudinal monitoring of dynamic vascular alterations associated with disease activity. Furthermore, its capability to visualize subcutaneous microvasculature suggests substantial potential for detecting pathological vascular remodeling in keloids.

Therefore, the present study aimed to longitudinally track keloid lesions using three-dimensional PAI during conservative treatment and to identify layer-specific vascular and oxygenation changes associated with local recurrence.

## 2 Materials and methods

### 2.1 Research design and patients

This prospective, observational, time-series, single-arm pilot study was approved by the Institutional Medical Ethics Committee (approval no. C1593) and registered with the UMIN Clinical Registry (UMIN000048978). Adult patients aged ≥ 18 years with keloids located on the trunk or extremities who were deemed eligible for local triamcinolone injections were enrolled. The exclusion criteria were as follows: pregnancy or breastfeeding, ongoing photodynamic therapy, presence of a cardiac pacemaker, an American Society of Anesthesiologists Physical Status (ASA-PS) classification [29] of IV or higher, active skin infection, or inability to maintain a stable imaging posture during repeated measurements. Written informed consent was obtained from all participants, and all data were anonymized before analysis. This study was conducted in accordance with the principles of the Declaration of Helsinki. Assessments were performed at baseline and subsequently at 1, 3, and 6 months following treatment.

### 2.2 Clinical evaluation

Clinical status and temporal changes in keloids were assessed using the Vancouver Scar Scale [30] and the Japan Scar Workshop Scar Scale (JSS) (Supplementary Information S1) [31]. Follow-up photographs were used to identify localized progression of keloids. Changes in color (red to white) and continuous regression (thick to flat) were defined as improvement, whereas newly developed hypertrophy, together with an increase in keloid height above the surrounding skin as measured by three-dimensional ultrasound (3DUS), was defined as recurrence. Newly appearing localized erythema without macroscopic thickening was classified as subclinical recurrence.

### 2.3 3DUS imaging

To complement photoacoustic imaging with structural soft-tissue information, 3DUS imaging was performed using the HI VISION Ascendus system (Hitachi Medical Corporation Co., Ltd., Tokyo, Japan). The system was equipped with a 9-MHz linear array transducer coupled to an electronically controlled motorized scanner [32]. The region of interest was defined as a rectangular area measuring 4 × 6 cm. B-mode images were continuously acquired at a scanning speed of 2 mm/s. The acquired image sequence was reconstructed into volumetric datasets with a voxel size of 0.03 × 0.03 × 0.1 mm using ImageJ software (National Institutes of Health, Bethesda, MD, USA).

### 2.4 Photoacoustic imaging

The PAI system (LUB-0, Luxonus Inc., Tokyo, Japan) used in this study has been described previously [26], [27], [28]. The system incorporates a hemispherical detector array (diameter, 60 mm; spatial resolution, approximately 0.2 mm) mounted within a water-filled tray integrated into a bed-type imaging platform. Laser excitation was generated using a Nd:YAG laser operating at a pulse repetition rate of 30 Hz. The output beam pumped two Ti:sapphire lasers to generate pulsed illumination at wavelengths of 756 nm and 797 nm. Dual-wavelength excitation was alternated to enable functional assessment of hemoglobin oxygenation, resulting in an effective repetition rate of 15 Hz per wavelength. Laser beams were merged into a single optical path and delivered through an optical fiber positioned beneath the detector array. The optical beam was expanded to uniformly illuminate the region of interest co-registered with 3DUS imaging while maintaining optical fluence below the maximum permissible exposure (MPE) defined by the American National Standards Institute (ANSI) safety standards (ANSI Z136.1) [33]. The MPE under our experimental conditions was calculated as 8.4 mJ/cm² per pulse at 756 nm and 10.2 mJ/cm² per pulse at 797 nm, assuming a repetition rate of 30 Hz. Because the two wavelengths were alternately delivered during imaging, the effective exposure per wavelength was reduced. However, to ensure safety, we adopted the more conservative MPE value at 756 nm as reference and maintained the laser fluence below this threshold throughout the study. During imaging, acoustic coupling was achieved using a transparent polyethylene terephthalate membrane and a water layer to ensure efficient transmission of optical and ultrasonic signals.

Three-dimensional datasets were reconstructed using PAT Viewer software (Luxonus, Tokyo, Japan), producing volumetric voxel data of 0.1 × 0.1 × 0.1 mm³ . Blood vessel oxygenation was estimated using S-factor mode [22], [25], calculated from wavelength-dependent photoacoustic signal intensity ratios reflecting differential hemoglobin absorption characteristics. The wavelength pair was selected such that 756 nm preferentially reflects deoxygenated hemoglobin absorption, whereas 797 nm corresponds to an isosbestic wavelength, enabling relative evaluation of oxygenation independent of total hemoglobin concentration.

The scanning field fully encompassed the predefined region of interest, and adjacent unaffected skin areas were simultaneously imaged as anatomical references.

### 2.5 Analyses

Purple ink skin landmarks were used to spatially align clinical photographs, 3DUS datasets, and 3D PAI datasets across longitudinal measurements. Based on baseline images, the mean depths from the epidermis of the superficial vascular layer and the subdermal venous network were approximately 1.0 mm and 3.0 mm, respectively. Accordingly, segmentation was performed by defining the superficial layer as the region within 2 mm from the epidermal surface. After segmentation of the superficial layer, the deep layer was automatically defined using a custom program that extracted the remaining image volume. Thus, while the superficial layer was defined manually, the subdermal layer was determined in an automated and reproducible manner. The images, separately rendered for the superficial and deep layers using the Viewer, were reviewed by experienced investigators to confirm the anatomical validity of the segmentation. Subsequently, vascular morphology and oxygenation were evaluated for each layer. Perforating arteries were defined as vertically oriented branching vessels [26], whereas the subdermal venous network was defined as horizontally oriented polygonal vascular structures [34]. Particular attention was directed toward regions demonstrating clinical or subclinical recurrence.

Quantitative assessments included clinical scoring, lesion size measurements on 3DUS—including major axis length, minor axis length, and keloid height above the surrounding skin—as well as S-factor analysis. S-factor values were obtained from superficial vessels and subdermal venous networks within keloid regions. Additional S-factor measurements were obtained from peri-keloid venous networks located ≥ 1 cm from lesion margins for longitudinal normalization. To minimize inter-session variability, three peri-keloid venous segments consistently identifiable across all time points were selected as internal controls. Relative S-factor values were calculated as the difference between each measured vessel and the median peri-keloid control value. This normalization enabled visualization of temporal oxygenation fluctuations independent of systemic variation. Relative S-factor maps were displayed using a continuous color gradient.

Correlations were evaluated among the following parameters: clinical scores (VSS and JSS), 3DUS–derived size measurements, relative S-factor values obtained from photoacoustic imaging (PAI), and the presence of hyperoxic superficial vessels. Changes between consecutive time points were categorized as increase, no change, or decrease. For relative S-factor values, “no change” was defined as a variation of less than 10%.

### 2.6 Statistical evaluations

Because this exploratory study represents the first longitudinal characterization of keloid vascular abnormalities using PAI and was not intended to evaluate treatment efficacy, sample size estimation based on statistical power was not performed. Normally distributed variables are presented as means ± standard deviations, whereas non-normally distributed variables are presented as medians with interquartile ranges (IQRs). Paired *t*-tests were used to compare relative S-factor inter-layer differences. Changes in parameters were coded as + 1 (increase), 0 (no change), and −1 (decrease), whereas the presence of hyperoxic superficial vessels was coded as 1 (present) and 0 (absent). Spearman’s rank correlation coefficient was used to assess associations between parameters. In addition, the Mann–Whitney *U* test was applied to evaluate the relationship between clinical scores and the presence of hyperoxic superficial vessels. Inter-session stability of the S-factor in the peri-keloid region across the four observation time points was assessed using the coefficient of variation (CV) and the intraclass correlation coefficient (ICC [1], [4]). A *p*-value < 0.05 was considered statistically significant.

## 3 Results

### 3.1 Patient characteristics

**Fig. 1:**
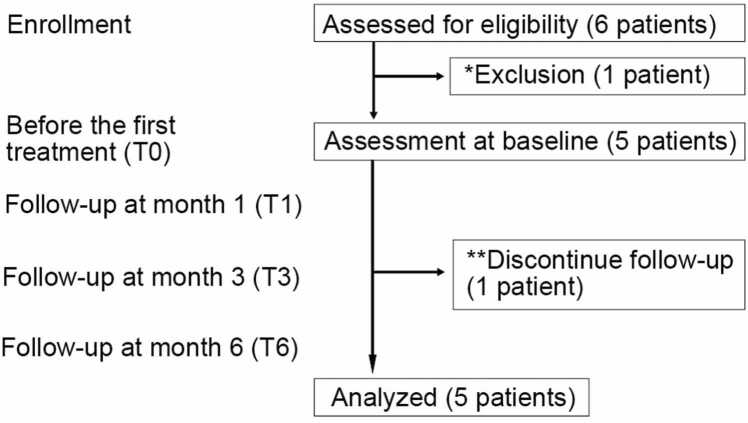
**Study flowchart.** T0, baseline; T1, follow-up at 1 month; T3, follow-up at 3 months; T6, follow-up at 6 months. *The patient was excluded because of an artificial shoulder joint and difficulty in maintaining the required posture on the day of imaging. **The 6-month examination was declined; imaging was conducted up to 3 months.

Patients were enrolled between December 2022 and July 2023. Of the six individuals who met the eligibility criteria and provided informed consent (Fig. 1), one patient was excluded on the day of the initial assessment because of inability to maintain the required posture during photoacoustic imaging acquisition. Ultimately, six keloids—three dumbbell-shaped, two oval, and one unclassified—from five patients (four men and one woman; median age, 39 years [IQR, 21–59]) were included in the study (Table 1).

**Table 1:** Clinical characteristics of keloids at baseline.

Case No.	Age (years)	Sex	BMI (kg/m^2^)	Anatomical Sites	Size (mm)	Causes	Shape
1	21	Male	18.3	Anterior chest	13 × 25	Trauma	Dumbbell-like
2	45	Male	23.0	Anterior chest	12 × 36	Acne	Dumbbell-like
3*	21	Female	23.0	Shoulder	5 × 12	Acne	Dumbbell-like
4	39	Male	22.3	Anterior chest	20 × 30	Acne	Oval
5*	21	Female	23.0	Shoulder	25 × 35	Surgery	Oval
6	73	Male	26.0	Upper arm	12 × 24	Vaccination	Unclassified

* Cases 3 and 5 involved the keloids from the same patient.

The keloids were located on the shoulders, upper arms, and anterior chest, with etiologies including vaccination, acne, and postoperative scarring following skin tumor excision. Five keloids from four patients completed all imaging sessions at baseline (T0), 1 month (T1), 3 months (T3), and 6 months (T6). One patient completed imaging up to T3 but missed the T6 session owing to neck pain. Imaging of healthy reference skin was not performed in one patient with two keloids to minimize procedural burden associated with prolonged imaging sessions.

### 3.2 Clinical characteristics

**Fig. 2:**
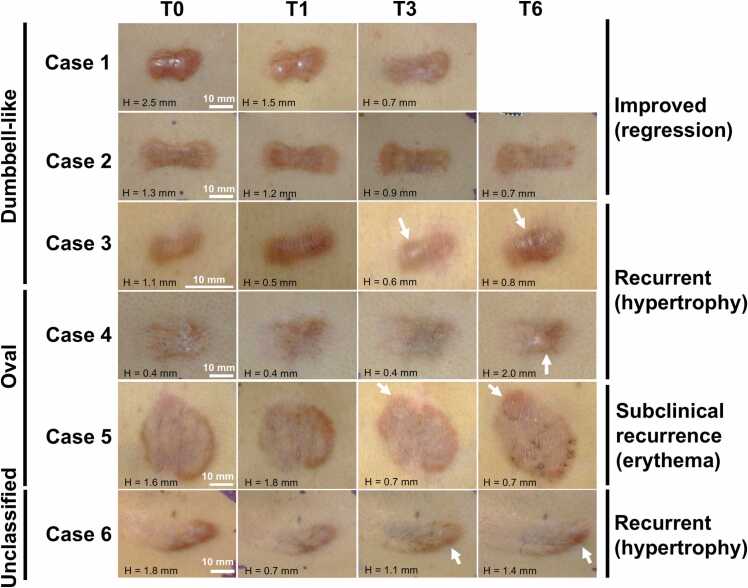
**Serial standardized photographs illustrating longitudinal changes in keloid appearance.** H indicates the height of the keloid above the surrounding skin, as measured by ultrasound imaging. For consistency, images for each case are scaled to the same size to facilitate direct comparison. Cases 1 and 2 demonstrate gradual regression during the follow-up period, whereas Cases 3–6 show localized erythema and/or hypertrophy during the latter half of the observation period (arrows).

Fig. 2 presents longitudinal clinical photographs of all six lesions together with keloid height measured using cross-sectional ultrasound imaging. During follow-up, two keloids (Cases 1 and 2) demonstrated gradual flattening accompanied by reduction of erythema, whereas four lesions (Cases 3–6) exhibited either overt recurrence or subclinical recurrence. Cases 3 and 4 developed central hypertrophy with increased erythema during the late observation phase (T3–T6). In contrast, Cases 5 and 6 demonstrated peripheral erythema and peripheral hypertrophy, respectively. Longitudinal changes in clinical scores and lesion dimensions are summarized in Supplementary Fig. S1. In Cases 1 and 2, Japan Scar Workshop Scar Scale scores decreased owing to improvement in erythema and pruritus. Conversely, Cases 3 and 4 showed increased scores associated with symptom worsening and increased lesion thickness. Morphometric analysis demonstrated heterogeneous temporal changes in major axis length, with elongation observed during regression (Cases 1 and 2) and shortening during hypertrophic progression (Cases 3 and 4). Minor axis length remained largely unchanged across all cases.

### 3.3 3DUS assessments

**Fig. 3:**
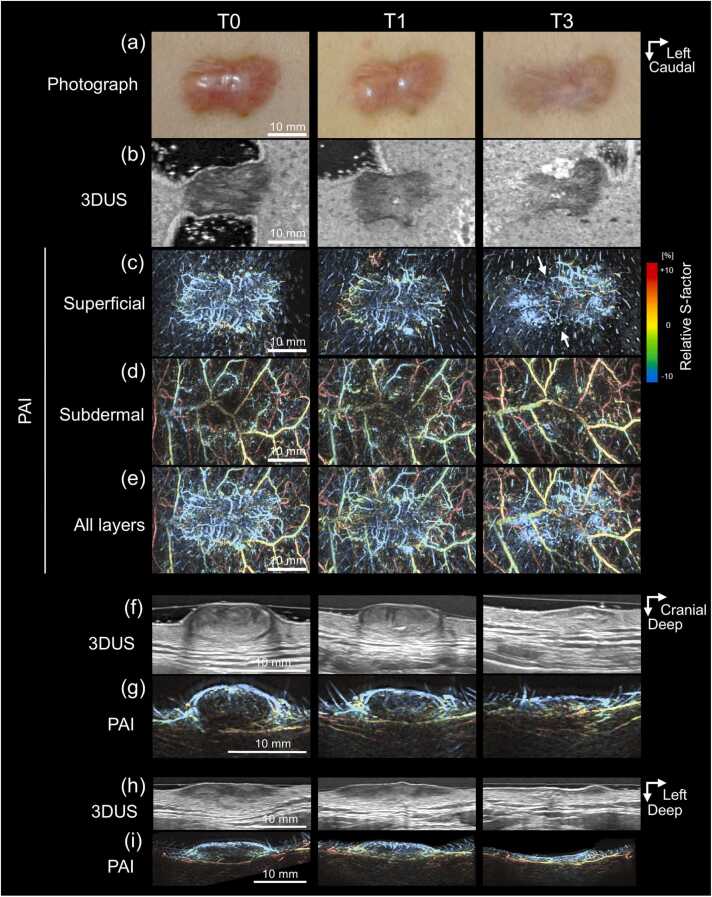
**Longitudinal changes in Case 1.** Before treatment, the keloid appeared elevated and erythematous and became flattened after treatment (a). Ultrasound images (b, f, h) visualize the keloid as a hypoechoic region, exhibiting an elliptical profile in the sagittal view (f) and a lenticular profile in the axial view (h). After treatment, the hypoechoic area decreases in size, and its boundary with the surrounding dermis becomes indistinct in the horizontal view (b). Photoacoustic images with color-coded relative S-factor values (c–e, g, i) reveal radial clusters of microvessels with uniformly low oxygen saturation surrounding the keloid surface. After treatment, the number of central microvessels decreases (arrows). The subdermal layer (d) contains a vascular network with heterogeneous oxygen saturation, and both the spatial distribution and oxygenation levels show minimal change after treatment.

**Fig. 4:**
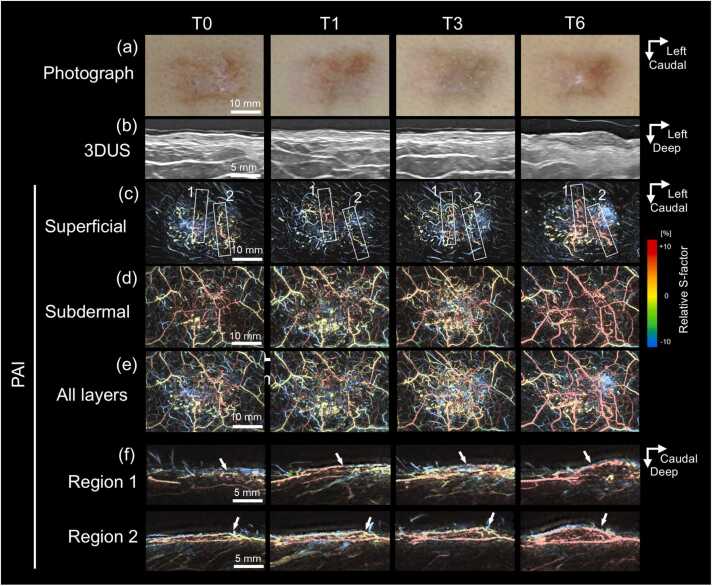
**Longitudinal changes in a recurrent Case 4.** Before treatment, the keloid exhibited a flat, oval morphology and subsequently recurred between T3 and T6 (a). Ultrasound images demonstrate thickening of the keloid profile at recurrence (b). Frontal-view photoacoustic images depict blood vessels and their relative S-factor using a color gradient across tissue layers (c–e). Aberrant tortuous microvessels are observed in the superficial layer (c). The subdermal layer contains a vascular network with heterogeneously elevated oxygen saturation (d, e). Sectional photoacoustic images (f), corresponding to rectangular regions 1 and 2 in (c), show upward displacement of superficial microvessels as the lesion thickened, with these vessels eventually overlying the recurrent lesion (arrows). At T6, the superficial microvessels exhibit increased oxygen saturation and demonstrate peripheral continuity with the subdermal vascular network.

**Fig. 5:**
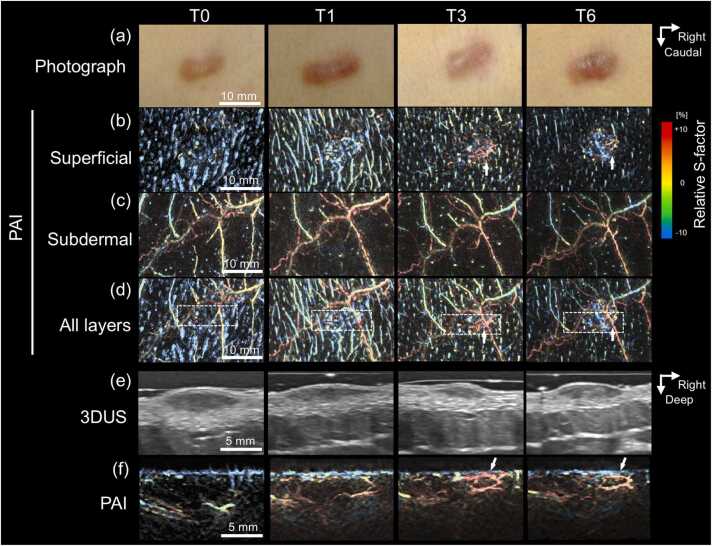
**Longitudinal changes in Case 3.** The keloid initially exhibited a small, oval morphology (a) with a lenticular profile on ultrasonography (e). The lesion flattened between T0 and T1; however, thickening was observed between T3 and T6. Photoacoustic images with color-coded relative S-factor values reveal no clusters of microvessels in the superficial layer (b), whereas several veins with heterogeneously elevated S-factor levels are present in the subdermal layer (c). After recurrence, thickened superficial microvessels with elevated oxygen levels are observed (b, d, arrows). Sectional images corresponding to rectangular regions in (d) demonstrate curved continuity between these superficial vessels and the subdermal vascular network along the keloid periphery (f, arrows).

Before treatment, 3DUS demonstrated keloids as well-demarcated hypoechoic lesions (Fig. 3b). Dumbbell-shaped lesions exhibited an elliptical configuration in sagittal views (Fig. 3f) and a lenticular configuration in axial views (Fig. 3h). The dermis underlying the lesions appeared concave at baseline (T0), suggesting mechanical compression by the keloid mass. Following treatment, lesion flattening was accompanied by reduced conspicuity of lesion–dermis boundaries and partial restoration of hypodermal morphology (Figs. 3f and 3h, T3–T6). In recurrent lesions, newly appearing hypoechoic regions were observed and progressively enlarged symmetrically during follow-up (Fig. 4b and Fig. 5e, T3–T6).

**Fig. 6:**
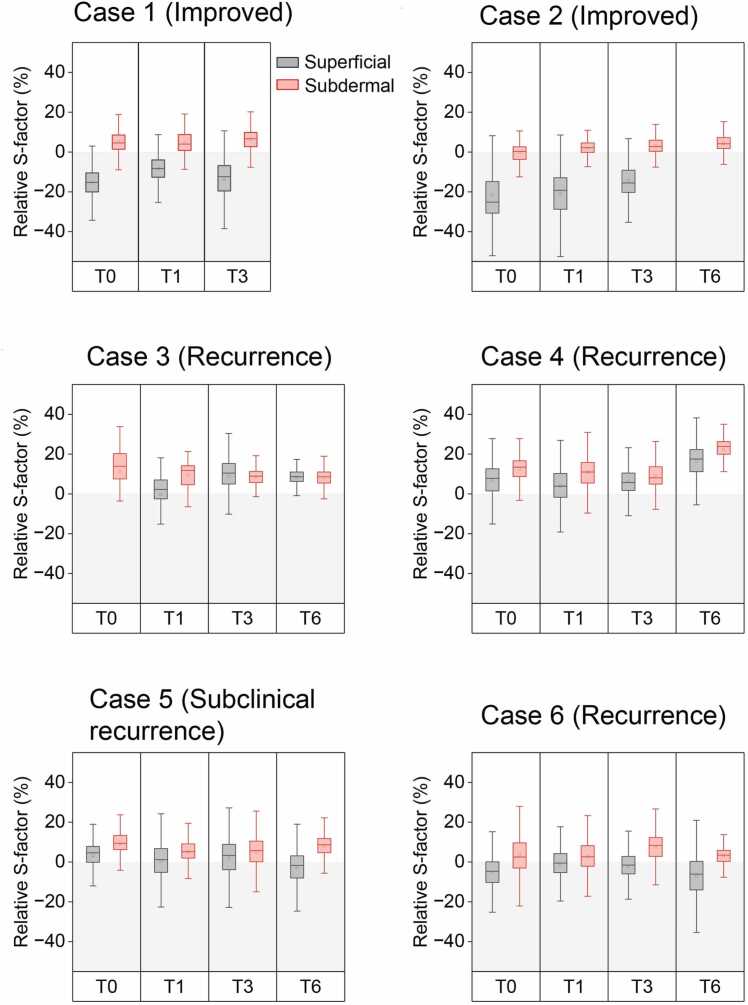
**Temporal changes in S-factor values.** Box-and-whisker plots illustrating temporal changes in relative S-factor values of superficial vessels (grey) and subdermal venous networks at the keloid (red) regions. In Cases 1 and 2, relative S-factor values of superficial vessels remain low, resulting in a marked disparity between superficial vessels and the subdermal venous networks. In contrast, Cases 3–6 show increased relative S-factor values in superficial vessels, leading to a reduced disparity between the two vascular components. Lines and squares within the boxes indicate median and mean values, respectively. Data for superficial vessels at T6 in Case 2 and T0 in Case 3 were unavailable owing to an insufficient number of analyzed vessels.

### 3.4 PAI assessments

PAI demonstrated layer-specific vascular abnormalities within keloid regions. In the superficial dermal layer, clustered microvascular structures were observed (Fig. 3c, Fig. 4c, and Fig. 5b). In contrast, the subdermal layer consistently exhibited polygonal venous networks showing heterogeneous moderate-to-high oxygenation levels (Fig. 3d, Fig. 4d, and Fig. 5c). The results of layer-specific S-factor measurements in keloids and peri-keloid regions are summarized in Supplementary Table S1. The intersession stability of the S-factor in peri-keloid regions was acceptable (mean CV, 4.0% [range, 0.5–6.7%]; ICC [1], [4], 0.70).

**Fig. 7:**
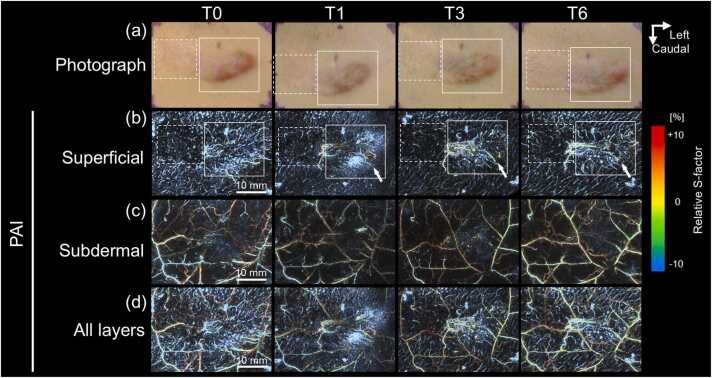
**Longitudinal changes in Case 6.** At baseline (T0), the keloid exhibited an elongated, slightly elevated morphology with erythema on the left side (solid boxes) and a flat, depigmented, scar-like lesion on the right (dashed boxes). After treatment, the erythema appeared to be resolved by T1 but recurred between T3 and T6 (a, arrowheads). Photoacoustic images with color-coded relative S-factor values (b–d) reveal concentric superficial microvessels surrounding the erythematous region (b, solid boxes). During the recurrence phase, hyperoxic microvessels emerge around the affected area (b, arrows), whereas no vasculature is detected within the scar-like lesion (b, dashed boxes). Elevated and heterogeneous S-factor values are consistently observed in the subdermal layer throughout the follow-up period (c).

Longitudinal PAI revealed distinct vascular evolution patterns between regressing and recurrent keloids. In regressing lesions (Cases 1 and 2), baseline imaging demonstrated radially arranged superficial microvessels exhibiting uniformly low relative S-factor values (Fig. 3c, T0). No structural continuity was observed between superficial hypoxic vessels and the underlying subdermal venous networks (Supplementary Video S1). During follow-up, progressive lesion flattening was accompanied by reduction in superficial vessel density, while low oxygenation levels persisted (Fig. 3c, T3). In contrast, recurrent lesions (Cases 3–6) demonstrated emergence of superficial microvessels with elevated relative S-factor values during recurrence phases. In Case 4, superficial vascular hyperoxia developed between T3 and T6 (Fig. 4c). Similarly, Case 3 demonstrated newly appearing hyperoxic superficial vessels localized to clinically recurrent regions (Fig. 5b). These vessels were radially distributed around elevated lesions, frequently forming hairpin-like configurations at lesion margins and exhibiting spatial continuity with hyperoxic subdermal venous networks (Fig. 4f and Fig. 5f; Supplementary Video S2). Comparable findings were observed in peripheral recurrence (Case 6), in which moderately oxygenated superficial vessels surrounded newly hypertrophic regions (Fig. 7).

Supplementary material related to this article can be found online at doi:10.1016/j.pacs.2026.100831.

The following is the Supplementary material related to this article Supplementary Video S1, Supplementary Video S2.

Supplementary Video S1 Video illustrating longitudinal alterations in the three-dimensional vasculature surrounding a regressing keloid (Case 1). Blood vessels are co-visualized with relative S-factor values using a color gradient. Hypoxic superficial microvessels are discontinuous from the hyperoxic subdermal venous networks (arrows)

Supplementary Video S2 Video illustrating the three-dimensional architecture of newly emerged microvessels at the site of recurrence in Case 3. Blood vessels are co-visualized with relative S-factor values using a color gradient. Hyperoxic superficial microvessels show continuity with the hyperoxic subdermal venous networks (arrows)

Table 2 summarizes temporal changes in clinical scores, 3DUS–derived size measurements, and PAI parameters in recurrent cases. Areas indicating exacerbation are highlighted in gray. The correlations among these parameters are presented in Table 3. The emergence of hyperoxic superficial vessels preceded keloid recurrence, defined as an increase in keloid height on 3DUS, and showed a strong association (Spearman’s correlation coefficient [*r*_*s*_] = 0.860). Although statistically significant, the associations between the presence of hyperoxic superficial vessels (PAI) and clinical scores were modest (PAI vs. VSS: *r*_*s*_ = 0.559, *U* = 8.0; PAI vs. JSS: *r*_*s*_ = 0.587, *U* = 6.5).

**Table 2:** Longitudinal changes in clinical scores, ultrasonographic measurements, and photoacoustic imaging findings.

Changes are shown relative to the previous time point. Arrows indicate direction of change: ↑, increase; ↓, decrease; →, no change. † Relative S-factor values are referenced to peri-keloid control vessels. No change was defined as a change of < 10% in relative S-factor values. 3DUS: three-dimensional ultrasonography; PAI: photoacoustic imaging. NA indicates data not available.

**Table 3:** Correlations among clinical scores, keloid height, and hyperoxic superficial vessels detected by photoacoustic imaging (PAI).

		**Parameter 1 vs. parameter 2**			
**Parameter 1**	**Parameter 2**	**Spearman’s correlation coefficient (*****r***_***s***_**)**	**One-tailed p-value**	**Mann–Whitney*****U***	**One-tailed p-value**
Vancouver Scar Scale (VSS)	Presence of hyperoxic superficial vessels (PAI)	-0.559	0.030	8.0	0.039
Japan Scar Workshop Scar Scale (JSS)	Presence of hyperoxic superficial vessels (PAI)	-0.587	0.020	6.5	0.031
Vancouver Scar Scale (VSS)	Increase in keloid height (US)	-0.481	0.055	—	—
Japan Scar Workshop Scar Scale (JSS)	Increase in keloid height (US)	-0.553	0.033	—	—
Increase in keloid height (US)	Presence of hyperoxic superficial vessels (PAI)	0.860	0.002	—	—

Changes in VSS, JSS, and keloid height were coded as + 1, 0, and −1 for increase, no change, and decrease, respectively.

The presence of hyperoxic superficial vessels on PAI was coded as 1 (present) and 0 (absent).

Given the small sample size, statistical analyses were exploratory.

## 4 Discussion

This study successfully delineated layer-specific three-dimensional vascular alterations associated with keloid regression and recurrence by combining PAI with three-dimensional 3DUS. The abnormal vascular architecture observed around keloids consisted of radially arranged superficial microvessels and a deep subdermal venous network. S-factor values in subdermal venous networks within keloid-affected regions were significantly higher than those in peri-keloid control regions. A notable difference in superficial vascular oxygenation was observed between clinically improving and recurrent lesions. In improving cases, superficial microvessels were hypoxic, resulting in a substantial interlayer S-factor gap between superficial and subdermal layers. In contrast, recurrent lesions exhibited the emergence of hyperoxic superficial microvessels at recurrence sites, which maintained continuity with hyperoxic subdermal venous networks. This feature was more strongly associated with increases in keloid height measured by 3DUS than with changes in clinical scores or relative S-factor values.

Vascular abnormalities in keloids have been extensively investigated using histopathological techniques. The literature contains conflicting reports describing both increased and decreased vascularity within keloid lesions, likely reflecting marked regional heterogeneity. Many studies have reported reduced vascularity in the keloid core [8], [35], [36], [37]. Increased expression of hypoxia-inducible factor–1α (HIF-1α) within the keloid core [37], together with frequent findings of vascular occlusion [8], [36], [38], [39], [40], supports the presence of a hypoxic microenvironment in this region. In contrast, peripheral and superficial regions demonstrate vascular abnormalities distinct from those of the central core. Reported features include increased vascular density suggestive of angiogenesis [35], [36], vertically oriented vessels, and vascular occlusion [9]. The dermis overlying keloids has also been shown to exhibit characteristic vascular alterations, including increased vessel numbers at approximately 400 μm depth [9], dense capillary loops [37], [41], and vessels arranged horizontally parallel to the epidermis [37], [42]. Liang et al. [9] further reported obliquely oriented superficial vessels along the keloid margin. Importantly, these histological observations are limited to single time-point assessment. Consequently, vascular transitions between progressive and regression phases remain poorly understood. Moreover, most histopathological studies evaluate vessels smaller than 50 μm, whereas vascular structures larger than 0.1 mm have rarely been assessed.

More recently, in vivo imaging modalities—including dermoscopy, optical coherence tomography, ultrasound, and photoacoustic imaging—have enabled visualization of vessels exceeding approximately 100 μm in diameter. Color Doppler ultrasound studies have demonstrated palisade-like vessels extending vertically from the subdermal vascular plexus into deeper keloid tissue [43], [44]. Although advances in high-frequency ultrasound have improved superficial vessel detection, Doppler ultrasound remains directionally dependent and exhibits limited sensitivity to flow oriented parallel to the skin surface.

PAI is a noninvasive imaging technique that uses near-infrared light to generate ultrasound signals from blood vessels [15], [45], [46]. Imaging depth depends on the penetration of near-infrared light; however, clear images can be obtained at depths of several centimeters in human tissue and approximately 10–20 mm in clinical settings [25], [26], [27]. The keloids examined in this study were less than 10 mm in thickness, making PAI suitable for comprehensive visualization from the lesion surface to the subdermal venous network located beneath the keloid. In contrast, PAM employs a focused ultrasound transducer for high-resolution imaging [15], [18], [47]. Optical-resolution PAM (OR-PAM) achieves a lateral spatial resolution ranging from submicron to a few micrometers, with an imaging depth of less than 1 mm, whereas acoustic-resolution PAM (AR-PAM) provides a lateral resolution of several tens of micrometers with an imaging depth of approximately 1–10 mm. By leveraging the complementary strengths of these modalities, it is possible to visualize both the fine vascular network within the dermal papillary layer and deeper subcutaneous vasculature. PAM has been widely used for real-time observation of microvascular dynamics, including vasoconstriction, and for detailed visualization of vascular structures in skin lesions [20], [48], [49], [50].

The PAI system used in the present study employs a hemispherical detector array that captures multidirectional ultrasound signals generated by thermoelastic vascular expansion. This detector geometry enables direction-independent reconstruction of three-dimensional vascular networks, providing comprehensive visualization of keloid vascular architecture. Furthermore, the non-invasive nature of PAI allows repeated examinations, enabling longitudinal assessment of vascular dynamics in benign proliferative disorders such as keloids, as well as malignant surface tumors including skin cancer [16], [19], soft tissue tumors [14], [51], and breast cancer [52], [53]. Three-dimensional vascular maps [28] allow detection of subtle regional vascular changes within identical anatomical locations over time.

A key finding of this study is the successful three-dimensional visualization of hyperoxic superficial microvessels associated with keloid recurrence. Although it remains unclear whether these vessels represent neovascularization or dilation of pre-existing vasculature within the papillary dermis, several observations provide mechanistic insight. In Case 3, newly developed superficial vessels emerged during recurrence, whereas in Case 4, moderately oxygenated vessels observed at T0–T1 became enlarged and hyperoxygenated during recurrence. Notably, these vessels consistently bypassed the keloid border and remained continuous with highly oxygenated subdermal venous networks. This anatomical continuity suggests a predominantly venous origin. The morphology of hyperoxic superficial vessels closely resembled hypoxic superficial vessels observed in improving lesions (Cases 1 and 2), suggesting a potential vascular transition from hyperoxic to hypoxic states during lesion maturation. Vascular stasis in the keloid periphery reported by Liang et al. [9] may contribute to this transition. In Case 6, moderately to highly oxygenated microvessels were detected in recurrent regions, whereas no superficial vessels were observed within regressed scar-like tissue, suggesting structural regression of superficial veins during lesion resolution.

The observed vascular hyperoxygenation indirectly suggests the presence of arteriovenous (AV) shunting within or surrounding keloid lesions. In such a mechanism, arterial blood may be diverted into venous circulation, resulting in secondary venous hyperoxygenation. Consistent with this hypothesis, Eura et al. [36] demonstrated dilated superficial vessels connected to internal thoracic perforator veins using contrast-enhanced computed tomography, supporting the existence of AV shunts within keloid tissue. Although AV shunts could not be directly identified in this study, they may exist within microvessels in or around keloid lesions. Such microvascular abnormalities may be better characterized using the micro-scale spatial resolution of PAM. In the future, longitudinal multimodal imaging combining PAI and PAM may provide deeper insights into the role of micro-AV shunts in keloid recurrence. Persistent hyperoxygenation within subdermal venous networks may likewise reflect sustained keloid activity.

Keloid progression is generally evaluated using scar-specific clinical scoring systems. In this study, increases in keloid height measured by 3DUS showed a stronger association with the presence of hyperoxic superficial vessels on PAI than with changes in clinical scores. Notably, these abnormal vessels appeared prior to clinically detectable recurrence. These findings suggest that longitudinal assessment using 3D PAI facilitates the early detection of microvascular changes associated with keloid recurrence.

This study has several limitations. First, the small sample size and lack of stratification by demographic and lesion-specific factors limit the generalizability of the findings. Second, the limited sample size precluded comparative analysis of temporal changes in relative S-factor values between the recurrence and regression groups. Third, the 6-month observation period may not capture the full course of keloid progression. Fourth, the spatial resolution of the PAI system (0.2 mm) limited the evaluation of finer capillary structures. Future integration of PAI with PAM may enable multiscale assessment of keloid vascular abnormalities. Fifth, manual segmentation of the superficial layer may affect reproducibility. Although our group has developed a body-surface extraction method based on a cloth simulation algorithm [54], it was not applicable in this study due to irregular skin contours associated with keloids. Further development of automated segmentation methods capable of handling complex surface geometries is warranted. Sixth, although the CV of the S-factor in internal control regions was acceptable, a system calibrated against an absolute oxygenation reference would further improve quantitative reliability.

In conclusion, three-dimensional photoacoustic imaging characterized dynamic vascular abnormalities associated with keloid regression and recurrence. When combined with 3D ultrasonography, this approach clarified the spatial relationship between vascular architecture and keloid morphology, demonstrating that the emergence of hyperoxic superficial microvessels preceded and was strongly associated with local recurrence. The subdermal venous network remained persistently hyperoxygenated, suggesting a potential role of AV shunting within or around keloid lesions. These findings highlight the potential of longitudinal PAI as a noninvasive modality for monitoring keloid vascular activity. Further development of advanced three-dimensional image analysis methods and quantitative oxygenation assessment based on absolute metrics, along with validation in larger clinical cohorts, will be necessary to confirm these findings and test the proposed mechanisms.

## Equal Contribution Statement

Aiko Makino and Susumu Saito contributed equally to this work. Aiko Makino was involved in the data acquisition, analysis and interpretation, and writing of the manuscript. Susumu Saito was involved in the study design, data acquisition, analysis and interpretation, and writing of the manuscript. Therefore, they should be considered co–first authors.

## Funding

This study was supported by a grant from the Japanese Agency for Medical Research and Development (AMED) (Grant Number: JP19he2302002).

## CRediT authorship contribution statement

**Aiko Makino:** Writing – review & editing, Writing – original draft, Visualization, Validation, Resources, Methodology, Investigation, Funding acquisition, Formal analysis, Data curation. **Susumu Saito:** Writing – review & editing, Writing – original draft, Visualization, Validation, Supervision, Software, Resources, Project administration, Methodology, Investigation, Funding acquisition, Formal analysis, Data curation, Conceptualization. **Ayako Takaya:** Writing – review & editing, Investigation. **Maria Chiara Munisso:** Writing – review & editing, Investigation. **Eiichi Sawaragi:** Writing – review & editing, Resources, Investigation. **Tomoko Kosaka:** Writing – review & editing, Investigation. **Naoki Morimoto:** Writing – review & editing, Supervision, Resources. **Aya Yoshikawa:** Writing – review & editing, Validation, Software, Resources, Methodology, Formal analysis, Data curation. **Hiroyuki Sekiguchi:** Writing – review & editing, Validation, Software, Resources, Methodology, Formal analysis, Data curation. **Yasufumi Asao:** Writing – review & editing, Validation, Software, Resources, Methodology, Formal analysis, Data curation. **Takayuki Yagi:** Writing – review & editing, Validation, Supervision, Software, Resources, Project administration, Methodology, Funding acquisition, Formal analysis, Data curation.

## Declaration of Competing Interest

The authors have no relevant financial or non-financial interests to disclose.

Four of the listed authors (Aya Yoshikawa, Yasufumi Asao, Hiroyuki Sekiguchi, Takayuki Yagi) are employees of the company that developed the photoacoustic imaging device used in this study. These authors have been engaged in multicenter collaborative research aimed at the clinical application of the device.

## Data Availability

Data will be made available on request.

## Footnotes

Appendix A Supplementary data associated with this article can be found in the online version at doi:10.1016/j.pacs.2026.100831.
