# Supplementary material for: Longitudinal three-dimensional photoacoustic imaging reveals hyperoxic perilesional microvasculature associated with keloid recurrence
Source: Photoacoustics. 2026 Apr 23;49:100831. doi: 10.1016/j.pacs.2026.100831 (PMC13139974; doi:10.1016/j.pacs.2026.100831)
Supplement: Supplementary file 3 — Supplementary material [file mmc1.docx]

**Supplementary information 1**

**Vancouver Scar Scale (VSS)**

The VSS consists of four variables:

- **Pigmentation**: 0 = normal, 1 = hypopigmentation, 2 = hyperpigmentation
- **Vascularity**: 0 = normal, 1 = pink, 2 = red, 3 = purple
- **Pliability**: 0 = normal, 1 = supple, 2 = yielding, 3 = firm, 4 = banding, 5 = contracture
- **Height**: 0 = normal/flat, 1 = <2 mm, 2 = <5 mm, 3 = >5 mm

**Japan Scar Workshop Scar Scale (JSS)**

The JSS consists of six variables:

- **Induration**: 0 = none, 1 = weak, 2 = mild, 3 = strong
- **Elevation**: 0 = none, 1 = weak, 2 = mild, 3 = strong
- **Redness of scars**: 0 = none, 1 = weak, 2 = mild, 3 = strong
- **Erythema around scars**: 0 = none, 1 = weak, 2 = mild, 3 = strong
- **Spontaneous and pressure-induced pain**: 0 = none, 1 = weak, 2 = mild, 3 = strong
- **Itching**: 0 = none, 1 = weak, 2 = mild, 3 = strong

Definitions of grading:

- **Weak**: Symptoms are localized to within one-third of the lesion or occur infrequently.
- **Mild**: Intermediate between weak and strong.
- **Strong**: Symptoms involve the entire lesion or occur frequently.


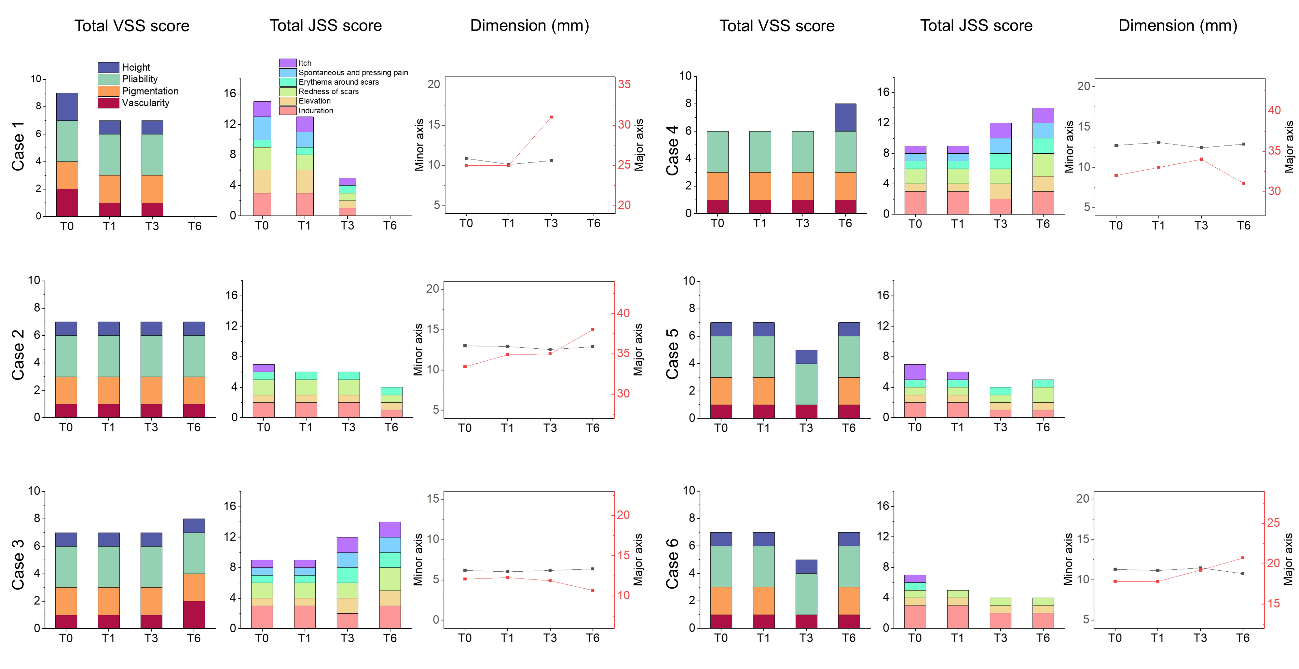


**Supplementary Figure S1.**

Bar graphs illustrate the changes in the clinical scoring systems (VSS and JSS scores) and lesion size in six keloid cases. In Cases 1 and 2, which exhibited regression during the observation period, the JSS scores decreased, whereas the major axis length increased. In Cases 3 and 4, which exhibited local hypertrophy between T3 and T6, both the VSS and JSS scores increased, accompanied by a decrease in the major axis length during that interval. In Case 5, size measurements were not performed because the keloid was circular in shape and lacked consistent landmarks for analyzing temporal changes.

**Supplementary Table S1: Measured S-factor values and longitudinal changes**

| Case | Region | The type of vessels |  | T0 | | T1 | | T3 | | T6 | | CV (%) |
| --- | --- | --- | --- | --- | --- | --- | --- | --- | --- | --- | --- | --- |
| 1 | Keloid | Superficial |  | 66.3 | (9.5) | 67.5 | (8.7) | 64.7 | (12.7) | NA |  |  |
|  |  | Subdermal |  | 86.1 | (7.1) | 83.0 | (7.9) | 83.8 | (7.1) | NA |  |  |
|  |  | Inter-layer disparity |  | -19.9 |  | -15.5 |  | -19.1 |  |  |  |  |
|  | Peri-keloid | Subdermal |  | 79.0 | (6.4) | 75.0 | (7.2) | 79.0 | (11.1) |  | (–) | 3.0 |
| 2 | Keloid | Superficial |  | 64.7 | (15.7) | 70.3 | (15.9) | 73.4 | (11.1) | No vessels | |  |
|  |  | Subdermal |  | 90.1 | (6.4) | 91.7 | (4.8) | 91.7 | (5.6) | 93.3 | (5.6) |  |
|  |  | Inter-layer disparity |  | -25.4 |  | -21.4 |  | -18.3 |  |  |  |  |
|  | Peri-keloid | Subdermal |  | 88.5 | (4.8) | 88.5 | (9.5) | 87.7 | (7.1) | 87.7 | (6.4) | 0.5 |
| 3 | Keloid | Superficial |  | No vessels | | 77.4 | (9.6) | 85.3 | (10.3) | 85.3 | (4.7) |  |
|  |  | Subdermal |  | 93.1 | (12.7) | 86.9 | (9.1) | 83.8 | (5.5) | 85.3 | (5.5) |  |
|  |  | Inter-layer disparity |  |  |  | -9.5 |  | 1.6 |  | 0.0 |  |  |
|  | Peri-keloid | Subdermal |  | 79.0 | (10.3) | 74.2 | (11.9) | 71.8 | (7.1) | 72.6 | (7.1) | 4.3 |
| 4 | Keloid | Superficial |  | 86.9 | (11.1) | 79.0 | (11.7) | 83.8 | (8.7) | 86.9 | (11.1) |  |
|  |  | Subdermal |  | 92.5 | (8.) | 86.1 | (10.3) | 86.1 | (8.7) | 93.3 | (6.4) |  |
|  |  | Inter-layer disparity |  | -5.6 |  | -7.1 |  | -2.4 |  | -6.4 |  |  |
|  | Peri-keloid | Subdermal |  | 78.2 | (6.4) | 73.4 | (6.3) | 73.4 | (7.1) | 66.3 | (8.7) | 6.7 |
| 5 | Keloid | Superficial |  | 84.5 | (7.9) | 73.4 | (11.9) | 86.1 | (12.7) | 75.8 | (11.1) |  |
|  |  | Subdermal |  | 89.3 | (7.2) | 77.4 | (7.2) | 88.5 | (10.3) | 86.1 | (7.1) |  |
|  |  | Inter-layer disparity |  | -4.8 |  | -4.0 |  | -2.4 |  | -10.3 |  |  |
|  | Peri-keloid | Subdermal |  | 82.2 | (11.9) | 73.4 | (7.1) | 78.2 | (11.1) | 75.8 | (9.6) | 4.8 |
| 6 | Keloid | Superficial |  | 72.6 | (10.3) | 86.1 | (9.5) | 81.0 | (8.7) | 81.4 | (12.7) |  |
|  |  | Subdermal |  | 79.8 | (12.7) | 89.3 | (10.4) | 90.9 | (9.6) | 89.3 | (5.6) |  |
|  |  | Inter-layer disparity |  | -7.1 |  | -4.8 |  | -9.9 |  | -8.0 |  |  |
|  | Peri-keloid | Subdermal |  | 77.4 | (7.2) | 82.2 | (11.9) | 81.4 | (7.9) | 85.3 | (5.5) | 4.9 |

Values are expressed as median (IQR).CV: coefficient of variation. NA indicates data not available.
